# Bringing Feedback in From the Outback via a Generic and Preference-Sensitive Instrument for Course Quality Assessment

**DOI:** 10.2196/resprot.4012

**Published:** 2015-02-13

**Authors:** Mette K Kaltoft, Jesper B Nielsen, Glenn Salkeld, Jo Lander, Jack Dowie

**Affiliations:** ^1^Research Unit for General PracticeDepartment of Public HealthUniversity of Southern DenmarkOdenseDenmark; ^2^School of Public HealthUniversity of SydneySydneyAustralia; ^3^Faculty of Public Health and PolicyLondon School of Hygiene and Tropical MedicineLondonUnited Kingdom

**Keywords:** medical education, nursing education, course assessment, course evaluation, student feedback, Internet, personalization

## Abstract

**Background:**

Much effort and many resources have been put into developing ways of eliciting valid and informative student feedback on courses in medical, nursing, and other health professional schools. Whatever their motivation, items, and setting, the response rates have usually been disappointingly low, and there seems to be an acceptance that the results are potentially biased.

**Objective:**

The objective of the study was to look at an innovative approach to course assessment by students in the health professions. This approach was designed to make it an integral part of their educational experience, rather than a marginal, terminal, and optional add-on as “feedback”. It becomes a weighted, but ungraded, part of the course assignment requirements.

**Methods:**

A ten-item, two-part Internet instrument, MyCourseQuality (MCQ-10D), was developed following a purposive review of previous instruments. Shorthand labels for the criteria are: Content, Organization, Perspective, Presentations, Materials, Relevance, Workload, Support, Interactivity, and Assessment. The assessment is unique in being dually personalized. In part 1, at the beginning of the course, the student enters their importance weights for the ten criteria. In part 2, at its completion, they rate the course on the same criteria. Their ratings and weightings are combined in a simple expected-value calculation to produce their dually personalized and decomposable MCQ score. Satisfactory (technical) completion of both parts contributes 10% of the marks available in the course. Providers are required to make the relevant characteristics of the course fully transparent at enrollment, and the course is to be rated as offered. A separate item appended to the survey allows students to suggest changes to what is offered. Students also complete (anonymously) the standard feedback form in the setting concerned.

**Results:**

Piloting in a medical school and health professional school will establish the organizational feasibility and acceptability of the approach (a version of which has been employed in one medical school previously), as well as its impact on provider behavior and intentions, and on student engagement and responsiveness. The priorities for future improvements in terms of the specified criteria are identified at both individual and group level. The group results from MCQ will be compared with those from the standard feedback questionnaire, which will also be completed anonymously by the same students (or some percentage of them).

**Conclusions:**

We present a protocol for the piloting of a student-centered, dually personalized course quality instrument that forms part of the assignment requirements and is therefore an integral part of the course. If, and how, such an essentially formative Student-Reported Outcome or Experience Measure can be used summatively, at unit or program level, remains to be determined, and is not our concern here.

## Introduction

Over several decades great efforts have been put into developing ways of eliciting valid and informative student feedback on courses they have taken in medical, nursing, and other health professional schools, and in continuing education and professional development. An important motivation has been “formative”, to help providers—teachers and related services—to improve what is offered. Their use in “summative” ways for administrative purposes, such as institutional promotion or staff evaluation, has increased greatly in recent years. However, whatever their motivation, items, and setting, the response rates have usually been low—only rarely above, or even approaching 50%—and potentially biased as a result. Many responses are produced cursorily, with little sense of engagement with a serious task. We see one of the main reasons for this as being its marginalized and optional status as “feedback” at the termination of the course, whether it is a day, or a year, long. Our goal is a new, generic, *course quality* assessment instrument and process, aimed at not only generating insights for the course provider into potential sources of improvement, but also, through the personalized and structured reflection it involves and encourages, enhancing the educational experience of the student. (In some countries and educational settings the term “evaluation” would be used instead of “assessment” in our context. We use the latter to embrace the former, for reasons that will become apparent.)

Why is a new instrument of this sort needed? A recent systematic review covers the vast literature on student evaluation and the instruments relating to it comprehensively and in depth [1]. While some of the numerous instruments are generic, applicable to all courses whatever the subject or focus, none produces a preference-sensitive index score, for example, an overall quantitative assessment that combines the individual student’s weightings for a set of quality criteria (dimensions) with their performance ratings for each of those criteria. Often course assessments are left as an unsynthesized profile of responses, but even where an index score is produced by some weighting procedure (including implicit equal weighting), the weights are not personalized. There is, therefore, a need for a generic and “dually personalized” measure of course quality, paralleling that in decision quality [2].

Beyond these two meta-criteria of genericness and preference-sensitivity, a third fundamental requirement is operational practicality. The instrument must be compatible with the time and other resources of students, on the one hand, and, if it were to be used summatively, capable of providing simple and actionable analyses by providers, on the other. But we see this practicality being established in the context of a substantially enhanced role for course assessment, which is now to be seen as a key source of the student’s benefit from the course. Without going so far as to suggest that, paraphrasing Socrates, "the unassessed course is not worth pursuing", we believe that student assessment of the quality of the course they are taking should be a formal part of it, not an optional, terminal add-on conceptualized merely as feedback. The idea is novel, but simply seeks to take advantage of, and gives direction to, the informal and unstructured judgements about, and reactions to, the course, that are occurring every moment the student is engaged with it.

## Methods

### Sources for the Course Assessment Instrument

A purposive survey of key references was sufficient to establish a comprehensive list of the attributes/criteria/dimensions that have been used in course assessment, evaluation, and feedback by students. Apart from the tabulation in Spooren [1], we consulted ten other sources: (1) Alderman et al [3], (2) Chalmers [4], (3) Coates [5], (4) Davies et al  [6], (5) Fontaine et al [7], (6) Kember and Leung [8], (7) Marsh and Roche [9], (8) Ramsden [10], (9) Richardson [11], and (10) Palmer [12].

**Figure 1:**
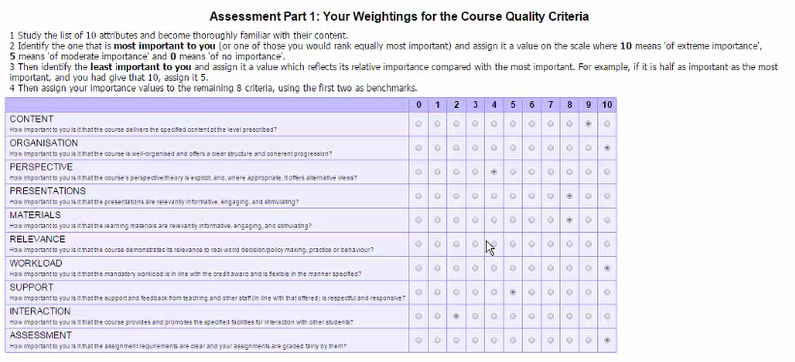
Screenshot from video on hypothetical student completing MCQ-10D.

Since the instruments reported in these studies were the result of extensive research and validation, the task in constructing a new instrument was not to add to the resulting list of criteria, but to reduce it to ten, the absolute maximum practical for routine use, especially in relation to criterion weighting. Both sets of responses are elicited on a 0 to 10 scale. The ten criteria would need definitions that were meaningful, in the sense that a single value on a 0 to 10 ratio scale could be provided as a response at both the weighting and rating stages. For weighting responses 0, 5, and 10 are labelled as “of no importance”, “of moderate importance”, and “of extreme importance”, respectively, and those values are labelled as performing “extremely poorly”, “moderately well”, and “extremely well” for course rating. It is made explicit in the instructions (Figure 1 shows this, later) that the scales are to be interpreted as ratio ones, as is necessary for the expected value calculation that produces the MyCourseQuality-10 Dimensions (MCQ-10D) index score (eg, 8 is to be twice as important as 4 on the weighting scale). (Some of the 10 criteria necessarily embrace the subcriteria and subsubcriteria included in more complex assessment instruments, and in these cases, the respondent's holistic high-level response will imply subweighting of these. For example, course materials may include different types of material, such as journal articles; videos; and applications for mobile devices.)

The final set of criteria for MCQ-10D was arrived at by considering the reported construct and content validity of the previous instruments, and maximizing comprehensiveness of coverage and conceptual independence within the constraint of 10 criteria. This necessarily involved making trade-offs based on value judgements, rather than purely statistical procedures.

The protocol for the piloting of the MCQ-10D enhanced course structure is organized using the Population, Intervention, Comparators, Outcomes framework [13].

### Population

Students in health professional education courses, for example, medical schools, subject to approval by the relevant bodies. (There are two approved pilot sites that are left unnamed in this publication).

### Intervention

Textbox 1 presents the full details of the MCQ-10D instrument. The Web-based survey in which it is embedded is live [14]. A video of a hypothetical student completing the survey is included as an appendix in this article (see Multimedia Appendix 1) (Figure 1) (Some of the questions supplementary to the instrument would be modified to suit the particular institution and course.).

MCQ-10D is completed in two stages, reflecting the aim to impact on the educational student experience from its beginning and throughout. Immediately prior to, or at the very start of the course, the student completes part 1, where they indicate the importance they personally assign to the 10 course quality criteria, on the 0 to 10 scale. (At both this point of time, and again in part 2 at the end of the course, they can indicate whether they had serious difficulty understanding any of the criteria and can leave comments on them.)

Students will be automatically reminded of the criteria at appropriate intervals (by email or announcements on their learning platform), for example, monthly, in courses lasting 8 weeks or more. In long courses, interim ratings may be appropriate, but these are not currently envisaged.

At the conclusion of the course, the student completes the lengthier part 2 of the assignment. In this, they provide their overall holistic assessments of course quality and satisfaction with it, followed by their ratings of the course on the MCQ-10D criteria, rephrased in the past tense.

Immediately after entering their ratings, students are presented with their MCQ-10D score in the Annalisa screen, which also displays the component ratings and weightings [15]. The score is the result of multiplying their ratings by their original weightings (normalized to add to 100%) and summing across all ten criteria. The student then has the opportunity to revise their weights, if they feel they are now different from the original ones they supplied (now visible to them), and thereby obtain a revised MCQ score. Next, they are able to see the partial contribution each criterion makes to the overall MCQ score, which will indicate to the providers the student’s views as to the possible sources of improved course quality. Note that, for each individual student, these will reflect his or her personalized weightings, as well as ratings. Finally, students are asked to reflect on whether explicit attention to course quality criteria via MCQ-10D has had an effect on their experience of the course, and to respond to other questions of a comparative nature. These questions are not part of the instrument and will necessarily vary with the course and its institutional setting. Those included on the Internet version represent one possibility.

It should be stressed that MCQ-10D can be implemented in many software programs, including macro-enhanced spreadsheets (eg, Excel or open source equivalents). Annalisa is an implementation of Multi-Criteria Decision Analysis, or, as in this use, Multi-Attribute Value Theory, and is simply one piece of software that facilitates the dynamic, interactive reweighting we regard as a key feature of the instrument.

From the outset, students are aware that MCQ-10D is a part of the assignment work for the course, with 10% of the course marks awarded for completion of both parts, the second of which is completed after they are aware of the marks they have received for the other 90% of the assignment work. They can therefore predict their grade with certainty before completing, or not completing, part 2 of the MCQ-10D assignment.

**MCQ-10D, with Internet heading and popup text (line 1) and Weighting and Rating questions (lines 2 and 3) for each dimension.**

CONTENT: scope of coverage and level of treatment

How important to you is it that the course delivers the specified content at the level prescribed?

To what extent do you think the course delivered the specified content at the level prescribed?

ORGANIZATION: clear structure and coherent progression

How important to you is it that the course is well organized and offers a clear structure and coherent progression?

To what extent did you find the course well organized and offered a clear structure and coherent progression?

PERSPECTIVE: explicit and offering alternative views where appropriate

How important to you is it that the course's perspective/theory is explicit, and, where appropriate, it offers alternative views?

To what extent did you find the course’s perspective/theory was explicit, and, where appropriate, it offered alternative views?

PRESENTATIONS: relevantly informative, engaging, and stimulating

How important to you is it that the presentations are relevantly informative, engaging, and stimulating?

To what extent did you find the presentations relevantly informative, engaging, and stimulating?

MATERIALS: relevantly informative, engaging, and stimulating

How important to you is it that the learning materials are relevantly informative, engaging, and stimulating?

To what extent did you find the learning materials relevantly informative, engaging, and stimulating?

RELEVANCE: to real world decision/policy making, practice, or behavior

How important to you is it that the course demonstrates its relevance to real world decision/policy making, practice, or behavior?

To what extent did you find the course demonstrated its relevance to real world decision/policy making, practice, or behavior?

WORKLOAD: appropriate to credit level and flexible

How important to you is it that the mandatory workload is in line with the credit award and is flexible as specified?

To what extent do you think the mandatory workload was in line with the credit award and exhibited the specified flexibility?

SUPPORT: from teaching and other relevant staff

How important to you is it that the support and feedback from teaching and other staff (in line with that offered) is respectful and responsive?

To what extent did you find the support and feedback from teachers and other staff (in line with that specified) was respectful and responsive?

INTERACTION: with other students

How important to you is it that the course provides and promotes the specified facilities for interaction with other students?

To what extent did you find the course provided and promoted the possibilities for interaction with other students that were offered?

ASSESSMENT: assignment requirements clear and mine graded fairly

How important to you is it that the assignment requirements are clear and your assignments are graded fairly by them?

To what extent did you find the assignment requirements were clear and your assignments were graded fairly by them?

### Comparators

Student reaction to the intervention will be gauged by responses to questions asking for their comparisons with the feedback system they conventionally experience. Also elicited will be their perceptions regarding the comparative effect of the intervention on their own educational experience, including the comparative quality and clarity of the opening course description.

No control group is envisaged, as it would be impractical, unethical, and possibly illegal. However, the group level results from MCQ-10D will be compared with the results from the standard feedback form that students are asked to complete anonymously in the institutions concerned.

Provider reactions to the intervention will be sought in a separate post course questionnaire, and interview/s which will involve requesting comparisons with their typical preparation of course descriptions, materials and presentations, their delivery of courses, and their perceptions of student performance and engagement.

### Outcomes

Student reactions to the experience are as specified under the subsection Comparators, immediately above. The MCQ score could be interpreted as a Student-Reported Outcome Measure or Student-Reported Experience Measure, analogous to a Patient-Reported Outcome Measure or Patient-Reported Experience Measure [16,17].

Provider/faculty reactions to intervention are as specified under the subsection Comparators, immediately above.

## Results

Initial piloting will occur in two courses during 2015, one in Australia and one in Denmark, with outcome results available by end of the year. However, other courses may be added on request.

## Discussion

###  Student Course Assessment as Graded Assignment

In certification settings, such as medical schools, experience shows that a task will rarely be undertaken if it is optional and does not count substantively to the course award. In many cases, simple (weighted, but ungraded) task completion will be an appropriate and sufficient requirement, as it will be in the case of MCQ-10D. It will effectively be a mandatory part of the assigned work, given a small, but finite weight (10%) in the final grade. Its satisfactory completion, defined purely technically, will add 10% to the student’s final mark. The actual course grade the student will receive is therefore predictable with certainty *before* the rating part of MCQ-10D is completed, or not.

If it is to be taken seriously, it is important that a course assessment instrument relates to the course as described in the rubric available to the student before enrollment (if it is an optional elective) or, at latest, at its commencement (if it is mandatory). The MCQ-10D instrument takes it for granted that the course has been designed to increase the person's degree of competency in relation to “knowing that”, or “knowing why”, or “knowing how”, or some combination of these. The content in terms of facts, principles, ideas, concepts, theories and techniques to be covered, the levels and depths at which they are to be (or can be) studied, the broad ways they will be presented and can be engaged with, the type/s of individual support and group interaction on offer, and the way/s competency will be assessed for certification purposes, are all to be spelled out explicitly in the course description. Secondary outcomes of the intervention are likely to be an improved quality of course preparation and greater precision and clarity in relation to the course’s aims and delivery methods, as well as wider potential benefits in curriculum development.

There is no provision *in the instrument* itself for the student to say they would have preferred the course to have been different from that offered. For example, to have some face-to-face sessions in a course clearly stated to be purely Internet, for basic material to be provided in what is clearly stated to be an advanced course, or for an “unflipped” course instead of the advertised “flipped” one. However, there is space in the survey, within which MCQ-10D is embedded, for this sort of comment, clearly differentiated and separated. We assume that alternative routes are available for forwarding such suggestions of changes to the course curriculum or rubric, some of which may involve increased resources being made available to the unit providers.

Students, like patients, are primarily persons, and should be treated as such. However, there is a central difference from medical or other health professional practice, in that the student is typically seeking certification from the provider for use in subsequent career situations. They are, in fact, purchasing the service which leads to that qualification, be it on a single unit of study or continuing development, or a complete award such as a degree, as well as gaining wider and noninstrumental benefits. “Person-centeredness” remains a key principle, but is necessarily different in the certification situation from that in a pure learning situation, since the awarding body has a duty of care beyond the individual. The resulting power relationship needs to be acknowledged throughout education, and especially in the seeking of feedback. In our proposal, the sequence of events ensures that the content of the student’s course assessment can have little influence on the grade awarded. Final submission of course ratings is essential to maximize marks, but can only occur after the student’s grade is predictable with certainty, because they know their marks for all their graded assignments.

As with all other aspects of the course, the student is made aware of this assignment requirement and consents to it by enrolling.

MCQ is explicitly designed for formative use at the course level. Appropriately interpreted, it could serve as one component of a multi-criterial summative assessment for other purposes, but introducing a dually personalized measure of quality as an integral part of the course will pose major challenges for those who seek aggregated “feedback” at unit, program, or higher levels.

### What Makes this Approach Different?

The key, almost paradigmatic, difference from previous instruments cited at the beginning of the paper is the use of the student’s importance weightings for the criteria. A second key difference is that the criteria presented are limited to ten as a matter of practicality, because of the need to make, or confirm, the explicit trade-offs among the criteria necessary in order to arrive at an overall index, and, hence, opinion as to the overall student-assessed quality of this course.

The individual student receives an immediate and personalized response to their course assessment as soon as their ratings are entered. This makes it somewhat rare among feedback instruments, which in most cases provide only delayed and aggregated information, if any.

Ideally, the instrument will also be completed by the course provider/s in the spirit of self-reflection and professional development. This would provide the basis of exploring dyadic concordances and discordances in an open manner at both overall and criterion-specific levels, and, hence, in relation to both course processes and course outcomes. Ultimately, only transparent discourse, taking place on a sound empirical basis and in a way that reflects student and staff heterogeneity, has the potential to deliver—as well as document digitally—person-centered education. However difficult it may be to implement an approach such as that represented by MCQ-10D within current systems, regulations, and resources, it represents the target to be aimed at from a long term and longitudinal perspective.

We have developed an Internet generic and preference-sensitive instrument for assessing course quality from the student perspective. It is intended to be practically useful for all parties who are willing to treat quality assessment as an integral part of a course, instead of as a marginal, terminal, and optional add-on as feedback, the focus of all previous instruments. Work is needed to test the instrument in a range of settings to establish its own quality and genericness, and how willing students and providers are to treat quality assessment as a process that both represents and creates educational added value.

This paper is a protocol to establish its feasibility and acceptability, and act as proof of method at the technical and organizational levels. It is to be piloted initially in courses in a medical faculty and a school for health professionals. We invite other health education providers to join in this piloting, using our software, and will be pleased to collaborate in proposals to translate the Internet instrument into other languages.

## Multimedia Appendix 1

Video of hypothetical student completing MCQ-10D.

## Abbreviations

MCQ-10D: MyCourseQuality-10 Dimensions
